# NCAPH Drives Breast Cancer Progression and Identifies a Gene Signature that Predicts Luminal A Tumor Recurrence

**DOI:** 10.21203/rs.3.rs-3231230/v2

**Published:** 2023-10-16

**Authors:** Marina Mendiburu-Eliçabe, Natalia García-Sancha, Roberto Corchado-Cobos, Angélica Martínez-López, Hang Chang, Jian Hua Mao, Adrián Blanco-Gómez, Ana García-Casas, Andrés Castellanos-Martín, Nélida Salvador, Alejandro Jiménez-Navas, Manuel Jesús Pérez-Baena, Manuel Adolfo Sánchez-Martín, María Del Mar Abad-Hernández, Sofía Del Carmen, Juncal Claros-Ampuero, Juan Jesús Cruz-Hernández, César Augusto Rodríguez-Sánchez, María Begoña García-Cenador, Francisco Javier García-Criado, Rodrigo Santamaría Vicente, Sonia Castillo-Lluva, Jesús Pérez-Losada

**Affiliations:** 1 Instituto de Biología Molecular y Celular del Cáncer (IBMCC-CIC), Universidad de Salamanca/CSIC, Salamanca, Spain.; 2 Instituto de Investigación Biosanitaria de Salamanca (IBSAL), Salamanca, Spain.; 3 Departamento de Bioquímica y Biología Molecular, Facultad de Ciencias Químicas, Universidad Complutense, Madrid, Spain.; 4 Instituto de Investigaciones Sanitarias San Carlos (IdISSC), Madrid, Spain.; 5 Biological Systems and Engineering Division, Lawrence Berkeley National Laboratory, Berkeley, CA, USA.; 6 Berkeley Biomedical Data Science Center, Lawrence Berkeley National Laboratory, Berkeley, CA, USA.; 7 Departamento de Medicina, Universidad de Salamanca, Salamanca, Spain.; 8 Servicio de Transgénesis, Plataforma Nucleus, Universidad de Salamanca, Salamanca, Spain.; 9 Departamento de Anatomía Patológica, Universidad de Salamanca, Salamanca, Spain.; 10 Servicio de Anatomía Patológica, Hospital Universitario de Salamanca, Spain.; 11 Servicio de Oncología, Hospital Universitario de Salamanca, Salamanca, Spain.; 12 Departamento de Cirugía, Universidad de Salamanca, Salamanca, Spain.; 13 Departamento de Informática y Automática, Universidad de Salamanca, Salamanca, España.

**Keywords:** Breast cancer, Luminal A subtype, NCAPH, LASSO, Genetic signature, Prognosis, Relapse-free survival

## Abstract

Despite their generally favorable prognosis, luminal A tumors paradoxically pose the highest ten-year recurrence risk among breast cancers. From those that relapse, a quarter of them do it within five years after diagnosis. Identifying such patients is crucial, as long-term relapsers could benefit from extended hormone therapy, whereas early relapsers may require aggressive treatment. In this study, we demonstrate that NCAPH plays a role in the pathogenesis of luminal A breast cancer, contributing to its adverse progression *in vitro* and *in vivo*. Furthermore, we reveal that a signature of intratumoral gene expression, associated with elevated levels of NCAPH, serves as a potential marker to identify patients facing unfavorable progression of luminal A breast cancer. Indeed, transgenic mice overexpressing NCAPH generated breast tumors with long latency, and in MMTV-*NCAPH/ErbB2+* double-transgenic mice, the luminal tumors formed were more aggressive. In addition, high intratumoral levels of *Ncaph* were associated with worse breast cancer evolution and poor response to chemotherapy in a cohort of genetically heterogeneous transgenic mice generated by backcrossing. In this cohort of mice, we identified a series of transcripts associated with elevated intratumoral levels of NCAPH, which were linked to adverse progression of breast cancer in both mice and humans. Utilizing the Least Absolute Shrinkage and Selection Operator (LASSO) multivariate regression analysis on this series of transcripts, we derived a ten-gene risk score. This score is defined by a gene signature (termed Gene Signature for Luminal A 10 or GSLA10) that correlates with unfavorable progression of luminal A breast cancer. The GSLA10 signature surpassed the Oncotype DX signature in discerning tumors with unfavorable outcomes (previously categorized as Luminal A by PAM50) across three independent human cohorts. This GSLA10 signature aids in identifying patients with Luminal A tumors displaying adverse prognosis, who could potentially benefit from personalized treatment strategies.

## Introduction

Breast cancer is currently the most frequently diagnosed tumor worldwide and the leading cause of cancer-related deaths in women(1). In recent years, assays have been designed to evaluate the risk of breast cancer relapse based on the expression of multiple genes, thereby enabling the selection of the best treatment option(2–8). Through these tests, intrinsic subtypes of breast cancer can be defined to enhance the ability to predict the evolution of this disease(5, 9–11).

One of the limitations of these signatures is that their long-term predictive ability has not yet been fully determined, particularly in luminal A tumors(10, 12). Luminal A tumors have the most favorable prognosis, with the highest disease-free survival (DFS) rate at ten years; however, it should be noted that 25% of these tumors that relapse do it within the first five years, and the relapse risk notably triples between the 5th and 10th years (13). Furthermore, luminal A tumors are most prone to recurrence beyond the 10-year follow-up mark (14). Indeed, metastasis may appear many years after the diagnosis of luminal A breast cancer and even decades after the removal of the primary tumor(15, 16). Additionally, treated luminal A tumors that eventually relapse may evolve into aggressive metastatic cancers(17). Therefore, it is paramount to identify luminal A tumors with a heightened relapse risk in the short to medium term, as these may require a more aggressive therapeutic approach. Similarly, it is essential to identify patients at an elevated risk of long-term recurrence to enable meticulous monitoring and potentially extend their secondary chemoprevention hormone therapy(14). Thus, there is a clear need to identify gene signatures for tumors already defined as luminal A that could distinguish those with potentially worse evolution in short-, medium-, and long-term follow-up. Indeed, including genes that are involved in the pathogenesis of luminal A tumors with poor evolution is likely to be useful for obtaining signatures that may more accurately predict prognosis(18). In this sense, luminal A tumors with a worse prognosis would exhibit higher genomic instability, including multiple modifications to the focal copy number(19) and alterations in genes involved in mitosis(20).

*NCAPH* encodes a member of the Barr protein family that contributes to the condensin complex. Condensins are large protein complexes that assemble interphase chromatin into chromosomes and organize their segregation during mitosis and meiosis(21). Two condensin complexes have been described(22), of which the condensin II complex is predominantly located in the nucleus during interphase and binds to chromosomes during mitosis(23–25). Conversely, the condensin I complex is located in the cytoplasm and binds to chromosomes after rupture of the nuclear membrane(26). However, a small fraction of condensin I has been detected in the nucleus during interphase, where it helps regulate gene expression and chromosome condensation(26). These two multiprotein complexes share two central subunits: the structural chromosome maintenance proteins SMC2 and SMC4. They also contain three non-SMC subunits: NCAPD2, NCAPG, and NCAPH in the condensin I complex and NCAPD3, NCAPG2, and NCAPH2 in the condensin II complex. The NCAPH and NCAPH2 subunits belong to the kleisin protein family(27–29), and mutations in the latter have been implicated in genomic instability(30).

Here, we demonstrate that NCAPH participates in the pathogenesis of breast cancer and in chemotherapy resistance *in vivo* and *in vitro*. MMTV-*NCAPH*^*ErbB2+*^ double-transgenic mice overexpressing NCAPH generated more aggressive breast tumors, and in a cohort of genetically heterogeneous transgenic mice generated by backcrossing, these tumors had a worse evolution and a poor response to chemotherapy. Moreover, using the least absolute shrinkage and selection operator (LASSO) multivariate regression model(31), we identified a group of 10 genes associated with high intratumoral NCPAH levels from this cohort of mice that formed a signature capable of defining patients with a worse evolution of luminal A tumors, better defining those who could benefit from more personalized treatment.

## Methods

### Patient samples and immunostaining

Human primary breast tumors were collected at the University Hospital of Salamanca (Salamanca, Spain), after the Hospital’s Institutional Ethics Review Board approved the protocols for the collection and use of patient samples. Written informed consent was obtained from all patients to conduct the study on these tumor samples. NCAPH expression was evaluated in a retrospective study of 28 human tumor samples by immunohistochemistry (IHC), eight of which were from patients who had a poor evolution and developed liver metastases, while the other 20 had a good evolution. IHC was performed automatically using the Bond Polymer Refine Detection kit (BOND III: Leica, Biosystems, Leica Microsystems), probing the sections for 60 min with an antibody against NCAPH (HPA0030008, Sigma Aldrich) diluted 1:100 and then counterstained with hematoxylin. Appropriate positive and negative controls were used.

*Fiji* software was used to analyze NCAPH expression in human breast tumors(32), sampling seven fields for each tumor randomly with a Leica ICC50 HD camera at 40x magnification under the control of the Leica Application Suite V3.7 software. The mean intensity (MI) of the epithelial cells was obtained and the reciprocal intensity (RI) was calculated in each field by subtracting the epithelial MI from the background(33). The normalized RI was obtained by dividing the RI by the background value, and the mean normalized RI of the seven fields was calculated for each patient.

### *NCAPH* transgenic mice

All mice were housed at the Animal Research Facility of the University of Salamanca and all procedures were approved by the Institutional Animal Care and Bioethical Committee. The mice were maintained in ventilated filter cages under specific pathogen-free conditions and fed *ad libitum*. MMTV-*NCAPH* mice were generated at the Transgenic Facility of the University of Salamanca. A 2,226 bp fragment containing the entire human *NCAPH* coding region was generated by PCR, cloned, and inserted into the *EcoRI* site of the pMKbpAII plasmid containing the Mouse Mammary Tumor Virus (MMTV) promoter. The construct containing *NCAPH* free of any vector sequence (BssHII fragment) was injected into fertilized oocytes extracted from FVB animals. Mice were screened for the presence of the transgene in the Southern blots of tail DNA digested with *XhoI*. The blots were hybridized with the same *XhoI* DNA fragment (658 bp) and confirmed by qPCR performed on mammary gland tissues of 3-month-old mice. Two founders (*NCAPH #1* and *NCAPH #2*) were obtained and bred, and their tail DNAs were genotyped using PCR. Three MMTV-*NCAPH* cohorts were generated: one cohort of nulliparous mice (N = 10) and two cohorts of parous mice that were pregnant twice: MMTV-*NCAPH* #1 (N = 29) and *NCAPH* #2 (N = 29). FVB/N-Tg(MMTVneu)202Mul/J mice carrying the *avian erythroblastosis oncogene B2/neuroblastoma-derived* (*ErbB2/cNeu)* protooncogene under the control of the MMTV promoter (MMTV-*Erbb2/Neu* transgene)(34) were obtained from Jackson Laboratory. A new cohort of mice was obtained by crossing MMTV-*NCAPH* and MMTV-*ErbB2* mice to obtain dual transgenic mice (N = 33).

### Backcrossing and F1 allografts

A genetically heterogeneous mouse cohort was generated by backcrossing two inbred strains as previously described (35). Briefly, we crossed the breast cancer-resistant C57BL/6 mouse strain (C57) with an *FVB/N-Tg(MMTVneu)202Mul/J* susceptible strain (FVB). F1-*Neu*^*+*^ males generated with the transgene were mated with FVB non-transgenic females to obtain a backcrossed cohort of MMTV-*ErbB2* mice (BX-*Neu*^*+*^) (N = 147). The concentration of tail DNA was measured using a Nanodrop ND-1000 Spectrophotometer and used for genotyping. (35)

Tumor cells from BX-*Neu*^*+*^ mice were transplanted into F1 female recipients and 100 μL of a single-cell suspension containing 2–5 ×10^6^ cells was injected into both inguinal flanks of each mouse. Each tumor was transplanted into two individuals (N = 125). Chemotherapy was initiated when the tumor diameter reached 12 mm by treating 58 mice with docetaxel (25 mg/kg; Taxotere, Sanofi Aventis) and 69 mice with doxorubicin (5 mg/kg; Farmiblastina, Pfizer), and each drug was injected intraperitoneally (IP). Docetaxel and doxorubicin were administered every 8 and 10 days, respectively, and the mice were sacrificed when the tumor reached 25 mm in diameter or two months after the end of the treatment.

### Histological analysis

Tumors and mammary glands were fixed in 4% paraformaldehyde (PFA: Scharlau FO) for 24 h at room temperature and washed in 70% ethanol before being embedded in paraffin for automated processing (Shandon Excelsior, Thermo). The samples were sectioned and stained with hematoxylin and eosin to evaluate their pathology under a microscope. Five photos (10x magnification) were taken randomly with a Leica ICC50 HD camera under the control of Leica Application Suite V3.7 software, quantifying the relative ductal area of the mammary glands. Image analysis was performed using *ImageJ*, selecting the ductal area (epithelial cells forming the duct) while excluding the adipose tissue and duct lumen. The ductal epithelial area was divided by the total field area to calculate the relative percentages, and the mitotic index of the tumors was defined as very high if there were more than eight mitoses at 40x magnification, high if there were between four and eight mitoses, moderate when there were between two and four mitoses, and low if there were less than two mitoses. A pathologist evaluated this parameter in the Pathology Unit of our Center.

### Immunostaining of mouse tissue

Immunostaining of mouse tissues was performed at the Pathology Unit of our Center. Mammary gland or tumor sections (3 μm) were deparaffinized and probed with a primary antibody against Ki-67 (MAD020310Q at a 1:50 dilution: Master Diagnostica) using Discovery ULTRA (Roche). The secondary antibody used was OmniMap anti-Rb horseradish peroxidase (HRP) (#05269679001, Roche), and Ki-67^+^ cells were quantified using Leica Application Suite V3.7 software with five selected areas on the slide at 20x magnification.

### Cell culture

MCF-7 and BT549 cells were grown in complete DMEM containing 4.5 g/l glucose and L-glutamine (Sigma Aldrich) at 37 °C in a humid atmosphere containing 5% CO_2_. The medium was supplemented with 56 IU/ml penicillin, 56 mg/l streptomycin (Invitrogen, Carlsbad, CA, USA), and 10% fetal bovine serum (FBS: LINUS #16sV30180.03), and all cell cultures were routinely tested for mycoplasma contamination. The cell lines were analyzed for authentication at the Genomics Core Facility at the Instituto de Investigaciones Biomédicas “Alberto Sols” (CSIC-UAM, Madrid, Spain) using STR PROFILE DATA, the STR amplification kit (GenePrint^®^ 10 System, Promega), STR profile analysis software GeneMapper^®^ v3.7 (Life Technologies), and a Genomic Analyzer System ABI 3130 XL (Applied Biosystems). See the **Supplementary Methods** section for procedures related to cell viability, soft agar assays, Boyden chamber cell migration assays, and qPCR assays.

### Protein analysis

Proteins were analyzed by western blotting as previously described (35). In brief, proteins were extracted from the tumors in RIPA buffer (150 mM NaCl, 1% (v/v) NP40, 50 mM Tris–HCl [pH 8.0], 0.1% (v/v) SDS, 1 mM EDTA, 0.5% (w/v) deoxycholate), whereas the proteins from the cell lines were extracted in TNES buffer (100 mM NaCl, 1% (v/v) NP40, 50 mM Tris-HCl [pH 7.6], 20 mM EDTA), both buffers containing protease and phosphatase inhibitor cocktails (Sigma‒ Aldrich; #P8340). The recovered proteins were quantified using a Bradford Protein Assay (Bio-Rad, #5000006), resolved by SDS-PAGE on 10 or 12% gels (Bio-Rad, #456–8085), and transferred to polyvinylidene difluoride membranes (Immobilon-P, Millipore). The membranes were probed with the following primary antibodies raised against NCAPH (1:10000, #TA303239: OriGene), cyclin D1 (1:1000, #sc8396: Santa Cruz Biotechnology), γH2AX (1:200, Ser139 #05–636: Millipore), pCHK1 (1:200, Ser345 #2348: Cell Signaling), tubulin (1:1000, DM1A; Sigma‒Aldrich; #T6199), HSP90 (1:1000, #515081: Santa Cruz Biotechnology), actin (1:1000, #A5441: Sigma‒Aldrich), pERK1/2 (1:1000, #9101: Cell Signaling), total ERK1/2 (1:1000, #4696: Cell Signaling), pAKT S473 (1:1000, #32581: Elabscience), and total AKT (1:1000, #30471: Elabscience). Antibody binding was detected using HRP-conjugated anti-mouse or anti-rabbit secondary antibodies (1:10,000 dilution; Amersham) and visualized by enhanced chemiluminescence (ECL, #170–5061: Bio-Rad). Images were acquired using an ImageQuant LAS 500 Chemiluminescence CCD camera (GE Healthcare Life Sciences).

### Inducible system for *NCAPH*

An inducible mammalian expression construct encoding NCAPH was obtained by cloning *NCAPH* into a pRetroX-Tight-Puro vector (Clontech #632104). The MCF-7 and BT-549 inducible *NCAPH* systems were generated by co-transfecting pRetroXTet-On Advanced with pRetroX-Tight-Puro-*NCAPH*. *NCAPH* expression was induced in cells after exposure to doxycycline (10 μg/ml).

### Identification of differentially expressed genes (DEGs) and functional enrichment analysis

The quality and quantity of the total RNA isolated from the cells were determined using an Agilent 2100 Bioanalyzer and NanoDrop ND-1000. Affymetrix GeneChip mouse gene 1.0 ST arrays were used, according to the manufacturer’s protocol. Gene expression data for mouse breast cancers are available from the Gene Expression Omnibus (accession number GSE54582). The differentially expressed genes (DEGs) between animals with *Ncaph* high and *Ncaph* low in the BX-*Neu*^+^ cohort were identified using Transcriptome Analysis Console (TAC) software, using a cutoff change (|logFC| > 2.0) and adjusted P ≤ 0.05. Gene Ontology (GO) pathway enrichment analyses were performed using the R package “clusterProfiler” (36, 37).

The DEGs obtained in BX-*Neu*^+^ mice were analyzed by LASSO regression using the R package “glmnet” (31), and the lambda value was determined by cross-validation to penalize collinearity among genes. The animals were divided into three groups (high-, medium-, and low-risk) and their scores were calculated using the following equation:

$$\text{Risk}\;\text{score=}\sum_{i=0\;\text{exp}i\times \text{ßi}}^{\text{n}}$$

Kaplan‒Meier lifespan analysis was performed to compare the prognostic differences between the three groups (R package: “survival” (38, 39), “survminer” (40)). For human analysis, we studied a combined patient database using GOBO (Gene Expression-based Outcome for Breast Cancer Online) (41), an online tool that downloads gene expression levels from a 1881-sample breast cancer dataset. We used the GSE1456, GSE2603, GSE6532, GSE3494, GSE4922, GSE6532, GSE7390, GSE11121, GSE12093, GSE2034, and GSE5327 databases to select luminal A samples (401 patients). We first conducted a univariate Cox regression analysis of DEGs and then selected candidate genes related to relapse-free survival (RFS) according to a criterion of *P value* < 0.25(42). The selected genes obtained in the univariate analysis were further analyzed by LASSO regression using glmnet, and the regression coefficient lambda was determined by cross-validation. Specifically, the GOBO cohort was randomly split into a training set (70%) to develop the model and a test set (30%) for validation. We then identified 10 genes that helped define disease prognosis using the LASSO model, which we referred to as the Gene Signature for Luminal A 10 (GSLA10). For the Kaplan-Meier analysis of RFS, the patients were divided into three groups (high-, medium-, and low-risk), and the score was calculated using the aforementioned equation to compare the prognostic difference between the three groups. The results were then verified using receiver operating characteristic (ROC) curve analysis(43). Finally, we validated our model using two independent databases, METABRIC (Molecular Taxonomy of Breast Cancer International Consortium) (718 luminal A breast cancer patients)(44) and The Cancer Genome Atlas Breast Invasive Carcinoma (TCGA-BRCA, 499 luminal A breast cancer patients)(45), both of which are available from cBioPortal (https://www.cbioportal.org/). In comparison with the Oncotype DX signature, we also constructed an Oncotype DX model based on 16 non-housekeeping genes in the Oncotype DX panel using the same training cohort (GOBO) (throughout this manuscript, we will refer to the 16-gene subset used in the Oncotype DX assay as ‘Oncotype’ for clarity and brevity). In both the training cohort (GOBO) and independent validation cohorts (METABRIC and TCGA-BRCA), we evaluated, validated, and compared the ability of GSLA10 and Oncotype to define RFS (GOBO) and DFS (METABRIC and TCGA-BRCA) in luminal A tumors. During independent validation, both the GSLA10 and Oncotype pre-trained models from GOBO were deployed without any modifications.

### Statistical analysis

Depending on the data distribution, we calculated either the Pearson or Spearman correlation coefficients or performed a Student’s t-test or Mann– Whitney U test to compare continuous variables between the two groups. ANOVA, or the Kruskal–Wallis test, was used to compare continuous variables across more than two groups. We used the Kaplan–Meier (KM) estimator and log-rank test to compare temporal variables. For contingency analysis, we used Fisher’s exact test to analyze 2 × 2 tables and the chi-square test in other cases. For more material and methods data, see the **Supplementary Methods** section.

### Availability of data and materials

All data generated or analyzed during this study are included in this published article (and its supplementary information files). The datasets generated and/or analyzed during the current study are available from the corresponding authors upon reasonable request. The datasets analyzed during the current study are available from GOBO (http://co.bmc.lu.se/gobo)(41), METABRIC (https://www.cbioportal.org/), and TCGA-BRCA (https://www.cbioportal.org/).

## Results

### *NCAPH* overexpression induces mammary gland hyperplasia and breast cancer in mice

**Fig. 1.**
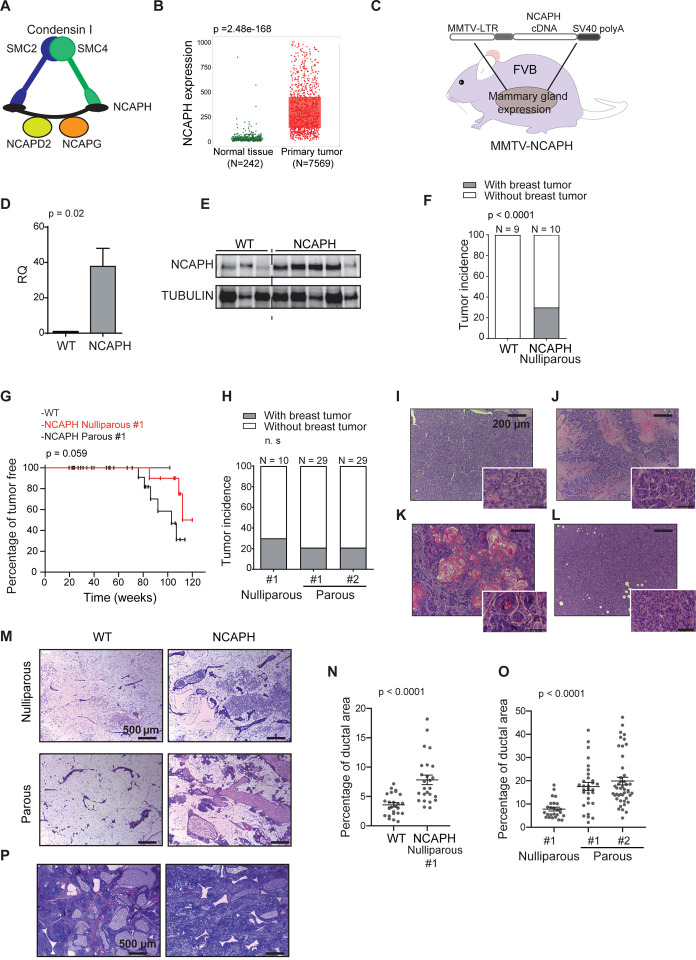
Transgenic mice that overexpress NCAPH develop breast cancer and mammary gland hyperplasias. **A**) Condensin complex 1, of which NCAPH forms a part. **B**) NCAPH is more strongly expressed in breast cancer than in normal mammary tissue. Analysis performed in the UALCAN portal using TCGA information: TPM, transcripts per million. **C**) The figure shows NCAPH overexpressed under the MMTV promoter to generate MMTV-NCAPH transgenic mice. **D**, **E**) Overexpression of NCAPH RNA in the mammary gland of transgenic mice quantified by QPCR (D) and the increase in protein seen in western blots (E). **F**) Incidence of breast cancer in mice overexpressing NCAPH after a 120-week follow-up. The controls were nontransgenic mice with an FVB genetic background. **G**) Tumor latency of MMTV-NCAPH#1 nulliparous and parous mice. Kaplan–Meier curves and log-rank test. **H**) Incidence of breast cancer in parous mice overexpressing NCAPH after a 120-week follow-up. **I**-**L**) Different histopathological patterns of breast cancer in MMTV-NCAPH transgenic mice (**Supplementary Table S1**). Glandular pattern (I); papillary pattern (J); squamous pattern with corneal pearls (K); solid pattern (L). **M**) Ductal hyperplasia in nulliparous and multiparous MMTV-NCAPH mice, with ductal dilation. **N**, **O**) Increased ductal parietal area of the mammary gland in MMTV-NCAPH nulliparous versus wild-type mice (N). The multiparous mice had a larger ductal parietal area than the nulliparous mice (O). N and O, T-test. **P**) Detail of a mammary gland from two MMTV-NCAPH mice with massive hyperplasia.

NCAPH is a constituent of the condensin complex, which facilitates the assembly of interphase chromatin into chromosomes and orchestrates their segregation during mitosis and meiosis(21) (Fig. 1A). Initially, we found that NCAPH overexpression was associated with poor outcomes in breast cancer(46, 47) (personal communications). This was determined through data mining analyses, specifically examining genes involved in mitosis and their correlation with adverse prognosis in breast cancer. We sourced our data from multiple databases: ETAM-158(48), GSE1456(49), GSE2034(50), GSE4922(51), and the dataset cited in(52) (**Supplementary Figure S1A**). Later, we identified an elevation in NCAPH levels in invasive ductal breast carcinomas compared to normal mammary tissues(53) (Fig. 1B). Consequently, this prompted us to investigate the potential involvement of NCAPH in the pathogenesis of breast cancer.

To determine whether NCAPH is a driver of breast cancer development, we generated transgenic mice overexpressing *NCAPH* under the control of the MMTV promoter (Fig. 1C), inducing NCAPH overexpression in the mammary gland (Fig. 1D, E). MMTV-*NCAPH* nulliparous mice developed breast tumors during long-term follow-up, with 30% of mice developing breast cancer after 120 weeks (Fig. 1F). Hence, the overexpression of *NCAPH* could drive breast cancer development. Oncogenes driven by the MMTV promoter are overexpressed during pregnancy because of promoter induction by gravidity hormones, resulting in more aggressive tumors(54). Indeed, the overexpression of NCAPH, regulated by the MMTV promoter in this transgenic line, resulted in breast tumor development with reduced latency post-pregnancy, suggesting a dose-dependent effect (Fig. 1G). Both *MMTV-NCAPH#1* and #2 mouse transgenic lines developed breast tumors after pregnancy, with no significant difference in tumor incidence (Fig. 1H). Infiltrating ductal carcinomas generated by *NCAPH* overexpression had a range of histopathological features, including breast adenocarcinomas with papillary differentiation, squamous differentiation, or a mesenchymal pattern (Fig. 1I–L and **Supplementary Table S1**).

Interestingly, although most nulliparous *MMTV-NCAPH* mice did not develop breast tumors after two years, they did show a significant increase in their ductal epithelial components. Indeed, *MMTV* promoter-driven overexpression of *NCAPH* produced hypertrophic mammary glands with marked ductal hyperplasia. The ducts were formed by a normal, single row of epithelial cells with no atypia and a benign aspect (Fig. 1M). However, there was a substantial increase in the number of ducts, increasing the total parietal ductal area (Fig. 1N, O). Notably, two additional mice that were not included in the comparison exhibited massive ductal and stromal hyperplasia in the mammary gland and the absence of fatty tissue (Fig. 1P). Together, these results indicate that *NCAPH* is oncogenic in breast tumors.

### NCAPH expression is associated with poor evolution and response to therapy in luminal A breast cancer patients

**Fig. 2.**
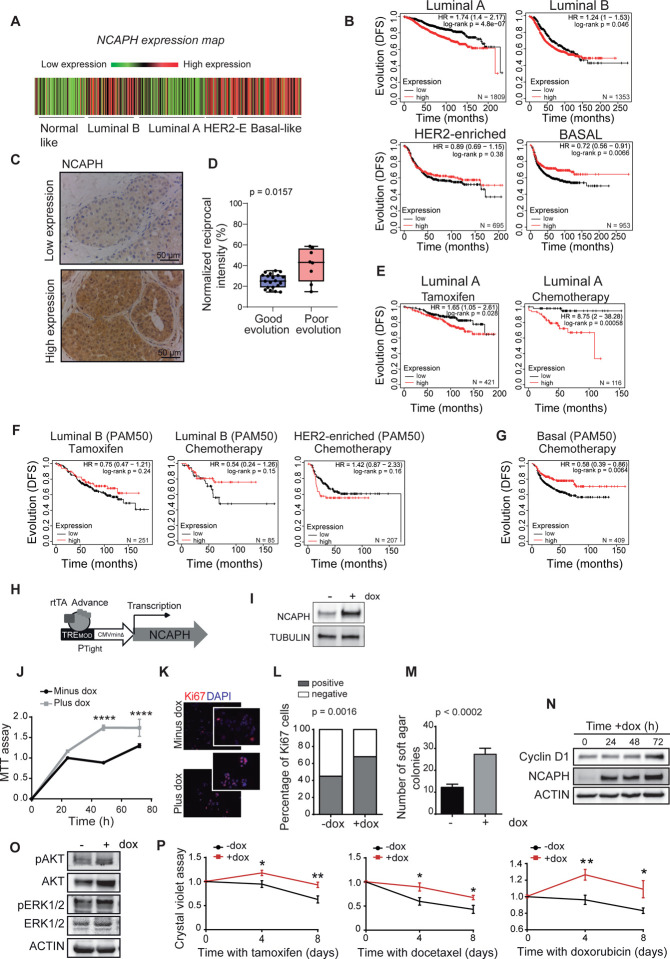
Intratumoral levels of NCAPH and evolution of breast cancer. **A**) Representation of NCAPH expression in the various intrinsic subtypes of breast cancer. Data were obtained from the bc-GenExMiner (64). **B**) Evolution (DFS, disease-free survival probability) of patients with different intrinsic subtypes of breast cancer, as defined by PAM50. **C**) Analysis of NCAPH expression by immunohistochemistry (IHC) in luminal A tumors. The panel shows an example of strong and weak expressions. **D**) Quantification of NCAPH levels determined by IHC in tumors with good or poor outcomes. **E**) Evolution (DFS) in luminal A patients (as defined by PAM50) treated with tamoxifen or chemotherapy. **F**) Patients with luminal B or HER2-enriched tumors were treated with tamoxifen and chemotherapy. **G)** Basal tumors (as defined by PAM50) were treated with chemotherapy. In A and D-F, the panels were generated with the Kaplan– Meier Plotter database, considering high and low intratumoral levels of NCAPH mRNA defined by the best cutoff(65). **H**) Diagram of the TET On system used to express NCAPH in response to doxycycline (dox). **I**) Expression of NCAPH in the MCF-7 cell line after a 24 h induction with dox. **J**) Assessment of cell proliferation in the MCF-7 cell line following NCAPH induction using the MTT test. **K**) Representative image of Ki-67 staining (red) and DAPI (blue) after a 24 h induction with dox in the inducible MCF-7 NCAPH cell line. **L**) Quantitative analysis of proliferative cells positive for Ki67 using Fisher’s exact test. **M**) NCAPH induction led to an increase in the number of MCF-7 colonies formed in soft agar. **N**) A Western blot illustrates the upregulation of cyclin D1 post-NCAPH induction. **O**) Analysis of AKT and ERK signaling pathways in the MCF-7 cell line post-NCAPH induction as evidenced by Western blotting. **P**) Increased cell resistance to various treatments, including tamoxifen, docetaxel, and doxorubicin, observed after NCAPH induction.

After demonstrating that NCAPH is involved in breast cancer development (Fig. 1) and is linked to poor prognosis in breast cancer (**Supplementary Fig. S1A**), we aimed to identify the specific intrinsic subtype of breast cancer where intratumoral NCAPH levels are associated with unfavorable outcomes. Interestingly, the most aggressive subtypes of breast cancer—specifically basal and HER2-enriched—exhibited the highest levels of NCAPH (Fig. 2A)(55). However, elevated NCAPH expression was also observed in patients from other subgroups. Consequently, we explored if NCAPH levels could differentiate between prognostically favorable and unfavorable forms within each intrinsic breast cancer subtype.

Breast cancer subtype classification and prognosis have been enhanced by gene signatures like PAM50 (Prediction Analysis of Microarray 50)(9). PAM50 provides a more precise intrinsic subtype classification than the St. Gallen approach, especially evident in the distinction between Luminal A and B subtypes, underscoring its superior molecular accuracy(56). Consequently, we employed PAM50 to explore whether distinct evolutionary groups emerge from intratumoral NCAPH expression under optimal classification conditions(57).

Although *NCAPH* expression was weak in the luminal A subtype compared with some other subtypes (Fig. 2A), we found that patients with high levels of *NCAPH* RNA were associated with poor evolution (*p* = 4.8 × 10^−7^). Paradoxically, basal-like tumors were associated with a good clinical evolution (*p =* 0.0066) (Fig. 2B). These results indicate the existence of a subpopulation of luminal A tumors that have poor evolution, which can be distinguished by their high intratumoral levels of *NCAPH*. We evaluated NCAPH levels by immunohistochemistry (IHC) in a cohort of patients with luminal A tumors (**Supplementary Table S2A**), and again, we confirmed the higher NCAPH protein levels in patients with a poor evolution (presence of liver metastases) than in those who evolved well in the 10-year follow-up (Fig. 2C, D). Moreover, NCAPH levels were not associated with other tumor characteristics such as grade, stage, histological subtype, or Ki-67 staining (**Supplementary Table S2B**). Significantly, high levels of *NCAPH* in luminal A tumors were associated with poor response to endocrine therapy and chemotherapy (Fig. 2E). This was in contrast with other intrinsic tumor subtypes that responded to therapy independently of *NCAPH* (Fig. 2F), except for basal tumors, which tended to respond well to chemotherapy when *NCAPH* levels were high (Fig. 2G).

To ascertain that elevated levels of NCAPH were associated with a diminished response to chemotherapy, we engineered an MCF7 luminal A breast cancer cell line wherein NCAPH overexpression could be triggered by doxycycline (Fig. 2H, I). The induction of NCAPH overexpression in MCF7 cells augmented their viability and proliferation (Fig. 2J–L), which correlated with the generation of significantly more colonies on soft agar (Fig. 2M) and elevated levels of cyclin D1 expression (Fig. 2N). The elevated levels of NCAPH were correlated with an increase in both total and phosphorylated AKT levels, which play a pivotal role in regulating various cellular processes including proliferation, survival, and cell growth (Fig. 2O). Remarkably, the upregulation of NCAPH led to partial resistance to therapy in MCF7 cells (Fig. 2P). We observed no discernible differences in cell viability in the basal BT-549 cell line following the induction of NCAPH expression or doxorubicin treatment (**Supplementary Fig. S2A**-**C**). Thus, high NCAPH expression in luminal A breast tumors is associated with a poorer prognosis and therapy response, indicating its potential as an identifier for high-risk luminal A tumors.

### intratumoral levels of NCAPH are associated with poor evolution in luminal HER2^+^ tumors

*NCAPH* levels were not associated with changes in the evolution of HER2-enriched tumors, which are ER-negative, as defined by the PAM50 (Fig. 2A, E). Since luminal HER2 tumors are not defined as an intrinsic subtype of breast cancer defined by PAM50, immunohistochemical classification was used to define the role of NCAPH in the prognosis of this tumor subtype (58).

**Fig. 3.**
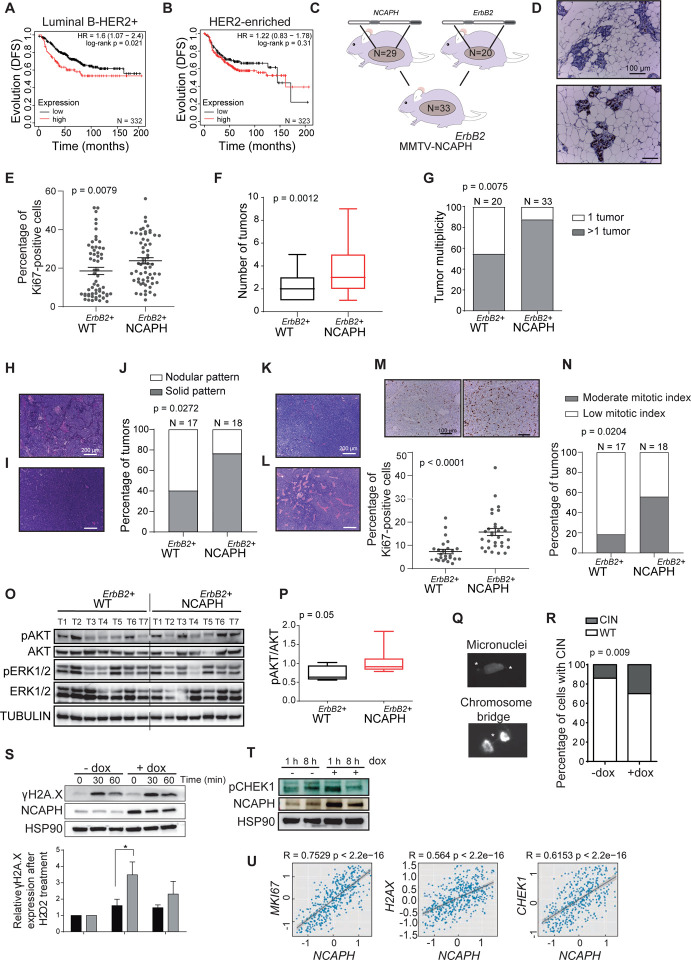
Effect of NCAPH expression on luminal ERBB2-positive tumors in vivo. **A**, **B**) Overexpression of NCAPH in patients with luminal ER-positive HER2^+^ breast tumors was associated with a worse evolution (A), while this association was not seen in HER2^+^ ER-negative tumors (B). Panels A and B were generated in the KM Plotter database(65). Evolution (DFS, disease-free survival probability). **C**) Scheme to generate double-transgenic mice MMTV-NCAPH^ErBb2^. **D, E**) Evaluation of proliferation by immunohistochemistry in nontumoral mammary glands of MMTV-ErbB2 and MMTV-NCAPH^ErBb2^ double-transgenic mice (D). Immunohistochemistry against Ki67 in a mammary gland of MMTV-ErbB2 and a double-transgenic one. Five mice were evaluated per group, and the field analysis is presented (E); Mann–Whitney U test. **F, G)** Quantification of tumor development. The double-transgenic mice developed significantly more tumors (F) and had greater multiplicity (G) than their MMTV-ErbB2 counterparts. **H**-**J**) Evaluation of the histopathological patterns, nodular (H) or solid (I). The solid pattern was more frequent in double-transgenic mice than in MMTV-ErbB2 mice (J): 10 mice per group were evaluated with a chi-squared test. **K, L**) Different degrees of vascularization in MMTV-ErbB2 and MMTV-NCAPH^ErbB2+^ double-transgenic mouse tumors. Hematoxylin-eosin staining of a tumor derived from an MMTV-ErbB2 (K) or an MMTV-NCAPH^ErbB2+^ double-transgenic mouse (L). **M**) Evaluation of tumor proliferation. Immunohistochemistry against Ki67 in a tumor from an MMTV-ErbB2 (left) or a double-transgenic MMTV-NCAPH^ErBb2^ mouse (right). Beneath the images, the quantification of the experiment is displayed. **N**) Quantification of the mitotic index: Chi-squared test. **O, P**) Assessment of key signaling molecules downstream of ERBB2 via Western blot analysis (O). Quantification of pAKT (P). **Q, R**) The induction of NCAPH increased genomic instability, as witnessed by the increase in the number of micronuclei and in the chromosomal bridge (CIN). Pictures illustrating the presence of micronuclei and chromosomal bridge (Q) and quantification of CINs (R). Fisher’s exact test. **S**) The increase in NCAPH expression was associated with genomic stress, as demonstrated by the increase in histone ɤH2AX expression after exposure to H_2_O_2_ and a 30- or 60- minute recovery time. **T**) Western blot showing changes in the expression of pCHEK1 after the induction of NCAPH. **U**) Correlation of NCAPH expression with that of Ki67, H2AX, and CHEK1 RNA in human samples.

Interestingly, high *NCAPH* levels were associated with poor clinical evolution of HER2^+^ luminal tumors (ER-positive), confirming that *NCAPH* did not influence the evolution of HER2-enriched tumors defined by IHC either (Fig. 3A, B). Thus, to investigate the role of NCAPH in the pathogenesis of luminal-HER2^+^ tumors, we crossed MMTV*-NCAPH* mice with MMTV-*ErbB2* transgenic mice that developed luminal ERBB2^+^ breast tumors(34) (Fig. 3C). An increase in epithelial proliferation was evident in the mammary glands of double-transgenic MMTV-*NCAPH*^*ErBb2+*^ mice relative to their MMTV*-ErbB2* counterparts, when Ki-67^+^ cells were stained by IHC and counted (Fig. 3D, E). Interestingly, the overexpression of *NCAPH*^*ErBb2+*^ in the mammary tissue of mice (MMTV-*NCAPH*^*ErBb2+*^) developed significantly more tumors than their MMTV*-ErbB2* counterparts (Fig. 3F, G), although no differences were observed in any other pathophenotypes of this disease.

Notably, while *NCAPH* transgenic mice developed distinct histopathological types of breast cancer (Fig. 1J–M), the MMTV*-NCAPH*^*ErBb2+*^ double-transgenic mice developed only infiltrating ductal adenocarcinomas, suggesting that the *ErbB2/Neu* oncogene exerts a dominant effect on the tumor phenotype. Therefore, the *ErbB2/Neu* oncogene appears to reprogram tumor differentiation so that instead of the histopathologically distinct tumors induced by NCAPH overexpression, the characteristics of infiltrating ductal adenocarcinoma predominated. Moreover, the double-transgenic mice predominantly developed infiltrating ductal adenocarcinomas exhibiting a solid histopathological pattern (Fig. 3 H–J), enhanced vascularization, and significantly heightened tumor proliferation compared to their MMTV-*ErbB2+* counterparts (Fig. 3K–M). Remarkably, tumors induced by NCAPH overexpression demonstrated a higher mitotic index (Fig. 3N).

The different histopathological behaviors of tumors from MMTV*-NCAPH*^*ErbB2+*^ double-transgenic mice prompted us to study molecules from some of the main pathways downstream of ERBB2/NEU. Intratumoral signaling was evaluated by assessing pAKT/pmTOR and pERK in western blots of seven tumors from each phenotype, and only pAKT levels were significantly higher in tumors from the double-transgenic mice than in those from the single-transgenic mice (Fig. 3O, P).

It is noteworthy that NCAPH overexpression in MCF7 cells also elevated the proportion of cells exhibiting chromosomal instability (CIN), wherein micronuclei and chromosomal bridges were identifiable (Fig. 3Q, R). Genomic instability triggers genomic stress (66, 67), as evidenced by the escalated levels of ɤH2AX and pCHEK1 following NCAPH overexpression and exposure to H2O2 (Fig. 3S, T). In human tumors, a robust positive correlation was discerned between NCAPH expression and proliferation markers (Ki67) or genomic instability markers (H2AX and CHEK1) (Fig. 3U).

The unique histopathological characteristics observed may emanate from elevated levels of NCAPH in the mammary gland, thereby exacerbating genomic instability and instigating secondary oncogenic events that target diverse tumor differentiation pathways(59).

Together, these findings propose that NCAPH overexpression in ERBB2+ tumors engenders more aggressive histopathology, solid tumors with high mitotic indices, and enhanced vascularization and cell proliferation. Thus, elevated levels of NCAPH promote a more aggressive form of breast cancer, potentially elucidating the adverse progression observed in luminal tumors.

### *NCAPH* expression is associated with poor breast cancer evolution in a genetically heterogeneous cohort of mice

**Fig. 4.**
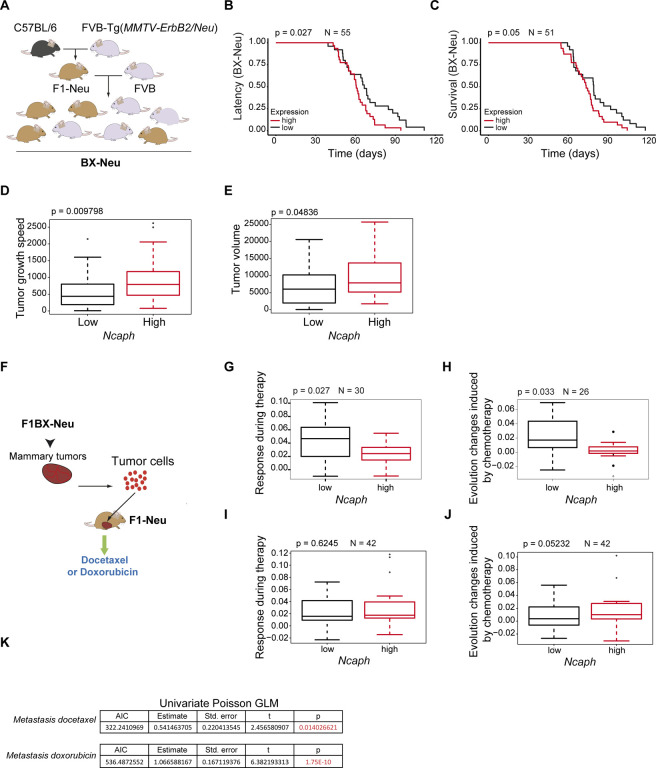
Intratumoral levels of Ncaph were associated with a worse evolution and response to chemotherapy in a cohort of mice generated by backcrossing (BX-Neu^+^). **A**) Scheme showing the generation of the BX-Neu^+^ cohort by backcrossing (see text). **B, C**) Decrease in tumor latency probability (B) and survival probability (C) in BX-Neu^+^ mice with elevated intratumoral levels of Ncaph: comparison of external tertiles. **D**, **E**) Rate of tumor growth and volume for tumors with high or low levels of Ncaph. **F**) Diagram showing the allogeneic transplantation strategy, passing tumors generated in BX-Neu^+^ mice into immunocompetent F1 mice. **G, H**) Tumors with elevated levels of Ncaph had a worse response to treatment (G) and after treatment (H) with docetaxel. **I, J**) There were no differences in tumor growth in F1 mice treated with doxorubicin during (I) or after treatment (J). D, E and G-J, Mann–Whitney U; B and C, Kaplan–Meier curves and log-rank test. **K**) Tumors with elevated levels of Ncaph were associated with significantly more lung metastases after treatment with doxorubicin and docetaxel (univariate Poisson GLM.)

Genetically heterogeneous mouse cohorts better reflect the heterogeneity observed in the human population, facilitating the identification of the genetic and transcriptomic determinants associated with disease evolution(35, 60, 61). Thus, to analyze the contribution of NCAPH expression to the heterogeneous evolution of luminal ERBB2^+^ breast cancer, we used a genetically heterogeneous cohort of MMTV-*ErbB2* transgenic mice generated by backcrossing (BX-*Neu*^*+*^ mice after that) (Fig. 4A). We crossed MMTV*-ErbB2* transgenic mice, which are on a susceptible FVB genetic background, with non-transgenic mice on a C57BL/6 genetic background, which is resistant to breast cancer development. We generated F1C57BL6/FVB MMTV-*ErbB2* mice (F1-*Neu*^*+*^ mice hereafter) that were backcrossed with FVB mice to generate BX-*Neu*^*+*^ mice, a cohort of mice with more varied breast cancer evolution than genetically homogenous mouse strains.

This BX*-Neu*^*+*^ cohort was used to evaluate whether intratumoral levels of *Ncaph* were associated with heterogeneous breast cancer evolution, demonstrating that high intratumoral *Ncaph* RNA levels were associated with shorter tumor latency and survival (Fig. 4B, C), faster tumor growth, and larger tumor volume (Fig. 4D, E). Thus, high levels of *Ncaph* are associated with poor evolution of luminal *Her2+/ErbB2*^*+*^ breast cancer in the cohort of BX-*Neu*^+^ mice.

Since elevated *NCAPH* levels mediate a poor response to chemotherapy in breast cancer patients and cells (Fig. 1G, 1L–N), we evaluated the response of tumors generated in BX-*Neu*^+^ mice to chemotherapy. Following the laws of transplantation in mice(62), breast cancer tumors that developed in the backcross cohort were transplanted into F1 mice, and their responses to anthracycline and taxane chemotherapy were evaluated. This strategy allowed us to evaluate the response of breast cancer to chemotherapy in an extracellular context as homogeneously as possible.

Thus, differences in treatment responses can be primarily attributed to differences at the cell-autonomous level (Fig. 4F). Tumors with high levels of *Ncaph* responded worse to docetaxel treatment, as reflected by a smaller reduction in tumor size (Fig. 4G).

In addition, the growth rate of tumors with high levels of *Ncaph* was faster, and their evolution was worse after chemotherapy than those that expressed *Ncaph* more weakly (Fig. 4H). However, *Ncaph* levels did not appear to influence the local response to doxorubicin (Fig. 4I, J). After chemotherapy with either doxorubicin or docetaxel, lung metastases were most evident in mice with high intratumoral *Ncaph* levels (Fig. 4K).

These findings suggest that high intratumoral levels of *Ncaph* are associated with resistance to breast cancer chemotherapy.

### A gene signature based on *NCAPH* expression defines poor tumor evolution in mice and humans

**Fig. 5.**
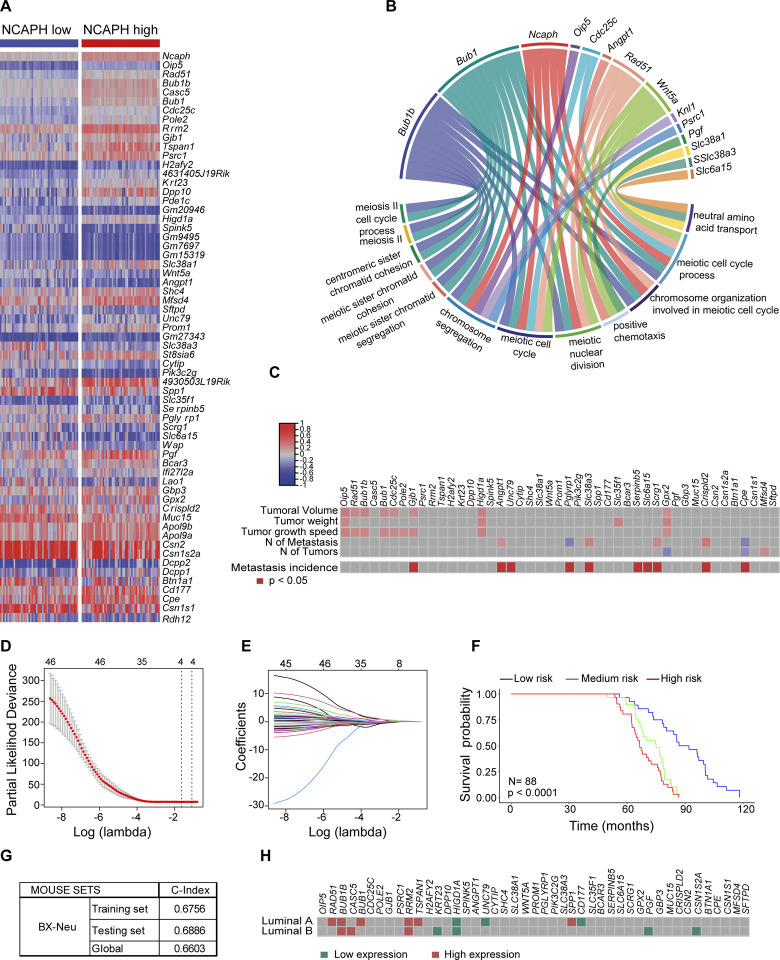
Identifying a gene signature associated with NCAPH in the MMTV-ErbB2 cohort of mice generated by backcrossing (BX-Neu^+^) recognizes the poor evolution in mice and humans. **A**) Heatmap showing the 64 differentially expressed genes between tumors with high or low intratumoral levels of Ncaph in BX-Neu^+^ mice, of which 45 were shared with humans. Extreme tertiles of Ncaph expression were taken to define the high and low levels of Ncaph. The criteria for underexpressed and overexpressed genes were a <−2-fold and a >2-fold change, respectively, and a value of p < 0.05 (**Supplementary Table S3**). **B**) Gene Ontology analysis shows the main biological functions in which the 45 genes of the signature participate (**Supplementary Table S4**). **C**) Some of the genes in the 45-gene signature were associated with the poor evolution of breast cancer in MMTV-ErbB2 mice generated by backcrossing. Correlation of the intratumoral levels of these genes with specific pathophenotypes of breast cancer in BX-Neu^+^ mice: Pearson’s test. The incidence of metastasis was evaluated with a chi-squared test. **D**-**G**) LASSO regression model that defines poor evolution in BX-Neu^+^ mice. The regression coefficient map of genes in the LASSO model, using cross- validation to select the optimal tuning parameter (λ). The dotted vertical lines were drawn at the optimal values using the minimum criteria and 1 standard error (SE) of the minimum criteria (the 1-SE criteria) (D). The graph shows the screening path of the LASSO regression model. Each curve represents a LASSO coefficient of the 45 prognostic genes, and the x-axis indicates the regularization penalty parameter. When the number of variables was 4, the partial likelihood deviation was at the minimum, corresponding to the minimum λ value (E). The LASSO regression model generated differentiates between low-, intermediate-, and high-risk BX-Neu^+^ mice in terms of survival probability and as defined by tertiles. Kaplan‒Meier curve and log-rank test (F). Goodness-of-fit measures for the generated LASSO models in the BX-Neu^+^ mouse cohort. Training, testing, and global model results (G). **H**) Some of the signature genes associated with high intratumoral levels of Ncaph in BX-Neu^+^ mice are also associated with a poor evolution of luminal subtypes of breast cancer (as defined by PAM50) in patients of the KM Plotter database (see **Supplementary Table S6**).

We examined the transcriptomic context in which *Ncaph* expression was associated with poor breast cancer evolution. The wide range of breast cancer evolution in backcross mice(35, 63) and the diverse expected patterns of gene expression(64) (Fig. 4) make this cohort an excellent tool for identifying transcripts associated with high levels of *Ncaph* expression and poor breast cancer evolution(35, 65). We identified 64 transcripts associated with high intratumoral *Ncaph* levels in breast tumors from the backcross cohort, of which 45 were shared with humans (Fig. 5A**, Supplementary Fig. S3A, B**, and **Supplementary Table S3**). The functions of this 45-gene signature were assessed using GO enrichment analysis.

Unsurprisingly, several genes associated with high *Ncaph* levels were involved in processes related to correct condensation and segregation of chromosomes during mitosis (Fig. 5B, Supplementary Fig. S3C, and Supplementary Table S4), and some were also correlated with poor breast cancer evolution in the BX-*Neu*^+^ cohort of mice (Fig. 5C). When we integrated several of these genes into a multivariate LASSO regression model to define poor tumor evolution in the BX-*Neu*^+^ mouse cohort (Fig. 5D, E and **Supplementary Table S5**), four genes were identified that were associated with poor survival in BX-*Neu*^+^ mice: *Oip5, Higd1a, Shc4,* and *Scrg1* (**Supplementary Fig. S3D-F**).

Interestingly, the intratumoral levels of certain genes identified in the heterogeneous BX-*Neu+* model, and associated with elevated levels of NCAPH, also correlated with adverse clinical outcomes (relapse-free survival [RFS]) in patients with the intrinsic luminal A subtype of breast cancer(57) (Fig. 5H and **Supplementary Table S6**).

In conclusion, elevated expression of NCAPH was linked to a set of gene transcripts. The intratumoral levels of these transcripts, akin to NCAPH itself, were also associated with the adverse progression of luminal breast cancer in both mice and humans.

### Identification of a genetic model associated with poor evolution in patients with luminal tumors

Despite having the best overall prognosis among the intrinsic subtypes, luminal A tumors display significant variation in prognosis. It is crucial to identify patients with poor prognoses for improved survival via initial therapeutic enhancements. High levels of *NCAPH* were associated with poor evolution, especially in luminal A tumors (Fig. 1D). Our study identified an array of genes that exhibited a notable correlation with elevated intratumoral levels of *Ncaph*, as depicted in Figure 5. We also found that some of these genes were associated with poor RFS in humans (Fig. 5H). Therefore, we used a penalized multivariate LASSO regression model to identify a gene signature that reflects the poor prognosis of luminal A tumors(31).

The LASSO regression model was generated from the GOBO database of 401 patients with luminal A-diagnosed breast cancers(41). This cohort was divided into a training set (70%) and a test set comprising the remaining 30% to generate a polygenic risk score.

First, bivariate analyses of the training set using Cox regression identified genes associated with poor evolution in terms of RFS (**Supplementary Table S7**). Later, genes with a *P* value < 0.25 were used to generate a polygenic risk score using the restrictive LASSO regression model(31).

**Fig. 6.**
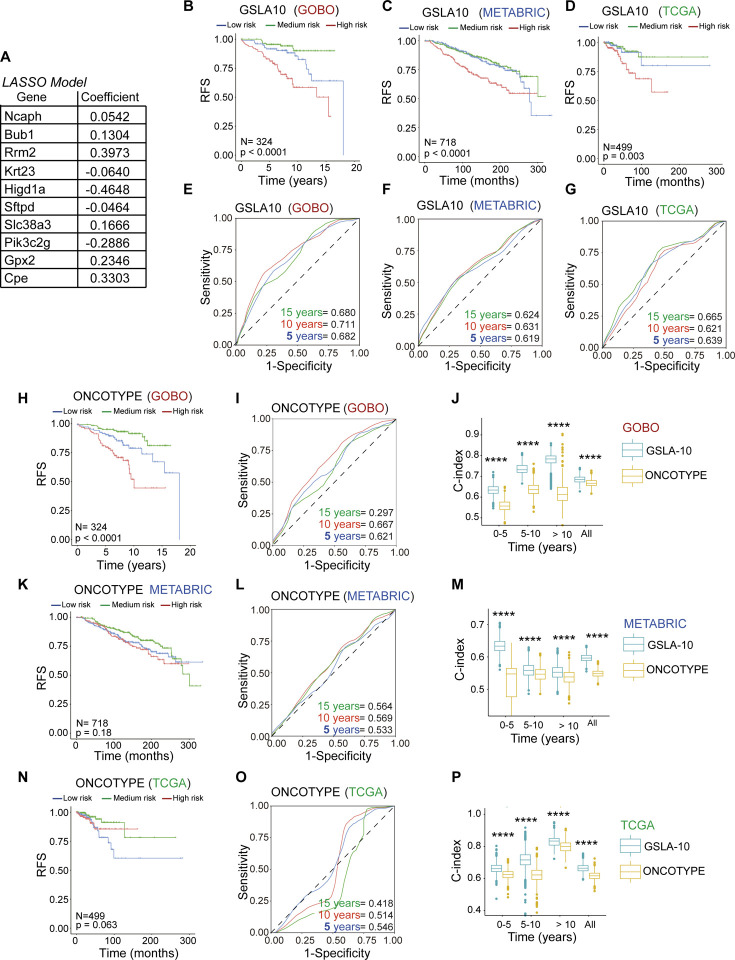
Comparative evaluation of GSLA10 and the Oncotype signature for prognostic determination in luminal A tumors. **A**) Hazard ratio illustration of each GSLA10 gene’s individual risk contribution, as implicated in the LASSO model from the GOBO cohort. **B**) Assessment of the GSLA10 model’s capability in delineating luminal A tumor progression via Kaplan‒Meier curves from the GOBO cohort. **C**) Validation of the robustness of the GSLA10 model for luminal A tumor progression definition using Kaplan‒Meier curves in the METABRIC cohort. **D**) Validation of the Oncotype model’s robustness in characterizing luminal A tumor progression with Kaplan‒Meier curves from the METABRIC cohort. **E**) ROC curves illustrating the prognostic prediction prowess of the GSLA10 model within the GOBO cohort. **F**) ROC curves demonstrating the GSLA10 model’s prognostic prediction in the METABRIC cohort. **G**) ROC curves of the GSLA10 model for prognosis prediction in the METABRIC cohort. **H**) The Oncotype model’s efficacy in defining luminal A tumor evolution was evaluated with Kaplan‒Meier curves in the GOBO cohort. **I**) ROC curves showcasing the Oncotype model’s ability to predict prognosis within the GOBO cohort. **J**) C-index comparison for prognostic prediction between GSLA10 and Oncotype models within the GOBO cohort. **K**) Validation of the Oncotype model’s robustness in characterizing luminal A tumor progression using Kaplan‒Meier curves in the METABRIC cohort. **L**) ROC curves of the Oncotype model for prognostic prediction in the METABRIC cohort. **M**) Comparison of the C-index for prognostic prediction between the GSLA10 and Oncotype models in the METABRIC cohort. **N**) Further validation of the Oncotype model’s robustness in defining luminal A tumor progression, as illustrated by Kaplan‒Meier curves from the METABRIC cohort. **O**) ROC curves of the Oncotype model for prognostic prediction in the METABRIC cohort. **P**) Comparative evaluation of the C-index for prognostic prediction between the GSLA10 and Oncotype models in the METABRIC cohort.

Thus, the LASSO model was developed, and ten genes were identified that define disease prognosis, which we referred to as the Gene Signature for Luminal A (GSLA10) (Fig. 6A**, Supplementary Fig. S4A, B**, and **Supplementary Table S8**). The prognoses in the training and testing sets and the global model were evaluated using the C-index (**Supplementary Fig. S3C, D**).

GSLA10 discriminated between low-(bottom 1/3 risk score), medium-(middle 1/3 risk score), and high-risk (top 1/3 risk score) RFS in patients (Fig. 6b). We validated our model in two independent patient cohorts, METABRIC and TCGA-BRCA, with 718 and 499 luminal A breast cancer cases, respectively. The C-index, AUC of the ROC curve, and log-rank *P*-value of the KM analysis were assessed when applying GSLA10 to the METABRIC and TCGA-BRCA cohorts, confirming the predictive capability of GSLA10 (Fig. 6 C, D and **Supplementary Fig. S4D**).

Additionally, we compared the ability of these signatures to define the prognosis of luminal A tumors in terms of RFS at different time points, evaluating the AUC for the first five years after diagnosis, between 5 and 10 years, and after 10 years in METABRIC and TCGA-BRCA (Fig. 6 E–G). The ROC curves indicated that GSLA10 can predict the risk of relapse in these patients at different time points. In addition, we constructed a risk model (Oncotype) based on 16 genes in the Oncotype (excluding five housekeeping genes). We provided a comprehensive comparison between GSLA10 and the Oncotype in terms of patient risk stratification and prognostic power in both the training cohort (GOBO) (Fig. 6H–J) and the independent validation cohorts (METABRIC and TCGA-BRCA) (Fig. 6K–M and N–P and **Supplementary Fig. S4E)**. The comparison further confirmed the robustness and superior prognostic power of GSLA10 over Oncotype in luminal A tumors.

In conclusion, the GSLA10 signature was associated with poor prognosis in luminal A patients. This model could help assess the prognosis of luminal A tumors and thus favor more personalized follow-up and therapy for patients with breast cancer.

## Discussion

In our study, we discovered a gene signature (GSLA10) linked to high intratumoral NCAPH levels and the unfavorable progression of luminal A breast tumors. The need for accurate identification of the prognosis of this tumor type is imperative because of differing initial treatment responses, including the potential inclusion of chemotherapy (66–69). Gene signatures, notably Oncotype DX, have been employed to identify potential chemotherapy beneficiaries among patients with ER-positive tumors(6, 70–72). Moreover, luminal A tumors exhibit the highest post-10-year relapse risk despite endocrine therapy, necessitating precise patient identification and potential extended hormonal treatment(73–75). Consequently, enhancing the prognostic precision of luminal A tumors is of crucial importance. In this context, our GSLA10 gene signature has shown superior prognostic power over Oncotype DX in both short- and long-term scenarios, suggesting its potential for tailoring luminal A patient treatment. Although GSLA10 demonstrated increased efficacy in predicting luminal A tumor prognosis, further studies are required to confirm whether this signature can reliably identify patients who may benefit from chemotherapy at diagnosis or from prolonged hormonal treatment.

We found that the overexpression of *NCAPH* is associated with poor prognosis, specifically in luminal A tumors and HER2^+^ luminal tumors. The specific association between high NCAPH levels and poor prognosis in luminal tumors may be related to the ability of condensin I complex to bind to ER enhancers. Indeed, condensins play an essential role in activating the expression of estrogen target genes through their activity at the transcriptional level(76). Moreover, the putative potentiation of estrogen signaling by NCAPH helps explain the hyperplasia observed in transgenic mice overexpressing NCAPH and their increased susceptibility to breast cancer development. This could also explain why NCAPH levels are not associated with the poor prognosis of breast tumors that do not express ER, such as HER2-enriched and basal tumors, as well as the participation of NCAPH in the poor clinical evolution of other hormone-dependent tumors, such as ovarian, endometrial, cervical, and possibly prostate cancers(77–82). Furthermore, luminal A tumors with worse evolution are thought to have higher genomic instability, alterations to P53(19), and overexpression of genes that regulate mitosis(20).

In the last five years, high levels of NCAPH expression have been associated with the pathogenesis and prognosis of several tumor types(77, 78, 80, 82–87), including hepatocarcinoma(88), lung tumors(89–91), and melanoma(92) and endometrial cancer(79). During the development of this project, two studies on NCAPH in breast cancer were published. The first study demonstrated that *in vitro*, NCAPH expression is elevated in MCF7 cells compared to the non-tumorigenic breast cell line, MCF10A(22). The second study revealed that downregulating NCAPH in MCF7 cells leads to a reduction in proliferation(47). In the present work, we substantially broaden the research on NCAPH’s role in breast cancer development. *In vitro*, we have demonstrated its involvement in viability, proliferation, increased genomic instability, alterations in cellular signaling at various levels, and resistance to multiple treatment modalities. Furthermore, we have introduced an *in vivo* transgenic mouse model that overexpresses NCAPH for the first time. Our findings suggest that NCAPH overexpression can eventually act as a primary oncogenic trigger for breast cancer. Notably, the overexpression of NCAPH leads to an enlargement of the non-tumorous breast’s glandular component, a recognized factor predisposing to breast cancer(93).

Our study also reveals a correlation between NCAPH and poor outcomes in HER2-positive luminal tumors in both humans and mice. In mice, this association results in breasts having an expanded glandular component and heightened proliferation—both of which are recognized as breast cancer risk factors(94–96). It was evident that HER2+ luminal tumors overexpressing NCAPH are more aggressive, characterized by rapid growth, increased proliferation, and a high mitotic index. Collectively, our discoveries substantially augment the current understanding of NCAPH’s role in the onset and progression of breast cancer. Given our findings that elevated NCAPH levels correlate with poorer outcomes in luminal Erbb2+/HER2/Neu tumors in transgenic mice, we identified a gene signature associated with high NCAPH levels. This was also conducted using a backcross cohort of mice with luminal Erbb2+/HER2/Neu tumors, as they epitomize an extension of the phenotypic presentation of breast cancer and its associated transcriptomics(50). In backcross models, a notable phenotypic variation in breast cancer is observed alongside a heightened transcriptomic variation, facilitating the association of both phenotype and transcriptome with their genetic regulation(60, 64, 65). Enrichment analyses revealed that some of the identified genes govern cell cycle progression and mitosis.

Interestingly, some of these genes were also associated with poor breast cancer evolution in BX-*Neu*^+^ mice, both individually and after the application of the LASSO regression model. It is important to note that some genes in this signature were individually associated with poor evolution of intrinsic luminal A subtypes in humans in the KM plotter database(57). Moreover, the application of some of these genes in a multivariate LASSO regression model identified luminal A tumors that relapsed in three human breast cancer datasets. It is plausible that patients with luminal A tumors, exhibiting high levels of NCAPH and displaying the associated signature could represent a distinct subgroup of tumors. This subgroup might exhibit an intermediate prognosis, positioned between those observed for the luminal A and luminal B subtypes. Further research is required to investigate this hypothesis and elucidate its potential taxonomic implications.

### Conclusions

In conclusion, we have demonstrated that NCAPH is involved in the pathogenesis of breast cancer. Moreover, an NCAPH-associated signature defines the evolution of the luminal A breast cancer subtype. The potential of GSLA10 to distinguish a specific cohort of patients with luminal A tumors, who might benefit significantly from intensified treatment protocols aimed at preventing relapse, is indeed stimulating. Certainly, the GSLA10 signature could serve as a diagnostic tool to identify patients with luminal A tumors who are at risk of poor prognosis. This insight may enable healthcare providers to devise personalized treatment strategies that are more efficacious for these patients.

## Translational Relevance

Luminal A breast cancer, while often less aggressive initially, can lead to metastasis long after primary treatment, with a notable fraction relapsing within five years. Identifying patients at risk of such aggressive recurrences is pivotal for treatment optimization. This study sheds light on the role of NCAPH in luminal A breast cancer development and progression, identifying it as a potential marker of poor prognosis. Moreover, our discovery of the GSLA10 gene signature, which outperforms established prognostic markers like Oncotype DX, underscores its potential utility in clinical settings. By distinguishing between patients who may benefit from aggressive treatment and those suited for prolonged hormone therapy, this research holds promise in significantly enhancing personalized treatment strategies for luminal A breast cancer patients, ensuring more targeted and effective therapeutic interventions.
